# Expression and localization of Sox10 during hair follicle morphogenesis and induced hair cycle

**DOI:** 10.7150/ijms.60728

**Published:** 2021-08-13

**Authors:** Jing Jing, Peng Xu, Jia-Li Xu, Yu-Xin Ding, Xiao-Shuang Yang, Xiao-Qin Jin, Li-Juan Zhou, Yu-Hong Chen, Xian-Jie Wu, Zhong-Fa Lu

**Affiliations:** 1 Department of Dermatology, the second affiliated hospital of Zhejiang University, Hangzhou 310000, China.; 2 Department of Neuro intensive Care Unit, the second affiliated hospital of Zhejiang University, Hangzhou 310000, China.; 3 Zhejiang Chinese Medical University, Hangzhou 310000, China.; 4 Department of Dermatology, Huashan Hospital Fudan University, Shanghai 200000, China.

**Keywords:** Sox10, hair follicle stem cell, morphogenesis

## Abstract

Sox transcription factors play many diverse roles during development, including regulating stem cell states, directing differentiation, and influencing the local chromatin landscape. Sox10 has been implicated in the control of stem/progenitor activity and epithelial-mesenchymal transition, yet it has not been studied in relation to the hair follicle cycle or hair follicle stem cell (HFSC) control. To elucidate the role of Sox10 in hair follicle cycle control, we performed immunohistochemical and immunofluorescence analysis of its expression during hair morphogenesis, the postnatal hair cycle, and the depilation-induced murine hair follicle cycle. During hair follicle morphogenesis, Sox10 was expressed in the hair germ and peg. In telogen, we detected nuclear Sox10 in the hair bulge and germ cell cap, where HFSCs reside, while in anagen and catagen, Sox10 was detected in the epithelial portion, such as the strands of keratinocytes, the outer root sheath (ORS) in anagen, and the regressed epithelial strand of hair follicle in catagen. These results suggest that Sox10 may be involved in early hair follicle morphogenesis and postnatal follicular cycling.

## Introduction

The mammalian hair follicle (HF) arises during embryonic development from coordinated interactions between the epidermis and dermis. The HF is a unique mini-organ that regenerates from the resting phase (telogen) to the growth phase (anagen), with rapid follicular keratinocyte proliferation and hair shaft elongation and thickening, followed by a regression phase (catagen) during its cycle 1. Epithelial-mesenchymal signalling and stem cell regulation play pivotal roles in hair follicle cycling, leading to the identification of several important biomolecules that control epithelial morphogenesis and growth.

Sox (Sry (sex determining region Y)-related HMG box) genes encode a family of transcription factors that are characterized by a conserved high-mobility group (HMG) domain that mediates their binding to DNA in a sequence-specific manner. *Sox* genes play key roles in embryonic development and are major determinants of stem cell behaviour, regulating cell fate decisions and maintaining cellular identity 2-3.

As a member of the Sox family, Sox10 regulates the expression of genes involved in the control of self-renewal and multipotency in both developmental and adult stem cells, such as embryonic neural crest cells and other adult stem cells or tissues, including inferior turbinate, periodontal ligament, mammary epithelium, and bone marrow 4-5. Furthermore, accumulating evidence suggests a role for Sox10 in several biological processes, including blood vessel tissue remodelling, proliferation, and differentiation, as observed in regenerating tissues and tumours 4.

Previous studies have focused on the role of the transcription factor Sox10 in regulating the expression of genes for soft tissue and epithelial tumors proliferation, invasion and metastasis 6-7. However, the role of Sox10 in hair cycling transitions and hair stem cell control remains poorly understood. In this study, we investigated Sox10 expression and localization in hair morphogenesis, the postnatal hair cycle and the depilation-induced hair cycle of C57BL/6 mice by immunochemistry and immunofluorescence.

## Materials and Methods

The methods of hair growth induction were approved by the Zhejiang University institutional review board on animal experimentation. We also carefully followed the Principles of Laboratory Animal Care (US Department of Health, Education and Welfare, NIH publication no. 85-23, revised 1985, Bethesda, MD).

### Induction of hair growth and harvesting of skin samples

For embryogenesis and postnatal study, sexually mature male and female C57BL/6 mice (Animal Center, Chinese Academy of Sciences, Hangzhou, China) were caged together at a ratio of 3:1 for 12 h, and the female vaginal suppository was checked the next morning. Mice with pessary were considered embryonic day 0.5 (E0.5). For the postnatal study, the birth date of the offspring and the date of skin sample harvesting were. More than three mice were sacrificed at each time point, E18.5, 1, 3, 12, 17, 19 and 25 days post birth.

For the depilation-induced hair cycle, syngeneic, 6-to-8-week-old female C57BL/6 mice (Animal Center, Chinese Academy of Sciences, Hangzhou, China) in the telogen phase of the hair growth cycle were selected. All mice were fed a standard diet and kept in a controlled environment in our animal facilities with an alternating 12-h day-night cycle. Anagen was induced in the dorsal skin of telogen mice by applying liquid rosin under anaesthesia according to previously described methods 8-11. Skin specimens were obtained at days 0, 3, 5 and 18 post depilation, representing the telogen, anagen II, anagen IV and catagen phases, respectively. Three mice per time point were sacrificed, and dorsal skin samples (0.3 cm × 0.5 cm) harvested from the depilated areas were fixed in 4% paraformaldehyde.

### Immunohistochemical and immunofluorescence study

For the immunohistochemical analysis, the dorsal skin in 4% paraformaldehyde was paraffin-embedded and cut into 5 μm-thick sections. Skin sections were dewaxed, microwaved in citrate-buffered saline and blocked at room temperature for 1 h in Tris-buffered saline supplemented with Tween-20 (TBST) with 5% bovine serum albumin. The skin sections were then incubated with 1/200 mouse anti-Sox-10 monoclonal antibody (sc-365692, Santa Cruz Biotechnology Inc., Santa Cruz, CA, USA) at 4 °C overnight as previously described 8-11. After washing, sections were labelled with horseradish peroxidase (HRP)-conjugated secondary antibodies (Zhongshan Goldbridge Biotechnology, Beijing, China) for 1-2 h at room temperature and visualized with DAB peroxidase substrate. Finally, sections were counterstained with haematoxylin (Zhongshan Goldbridge Biotechnology, Beijing, China).

For immunofluorescence analysis, after incubating with the blocking buffer, sections were incubated overnight at 4 °C with 1/200 mouse anti-Sox-10 monoclonal antibody (sc-365692, Santa Cruz Biotechnology Inc.), followed by washing three times with TBST. The sections were then incubated at 37 °C for 1 h with 1/1000 Alexa Fluor®488 conjunctated goat polyclonal secondary antibody to mouse IgG (Abcam, ab150113, USA). Finally, the sections were incubated in 0.1% DAPI (Sigma, USA) at room temperature for 5~10 min as a nuclear tracker. After washing in water, NanoZoomer S60 C13210 and NanoZoomer 2.0 HT digital pathology slide scanner (Hamamatsu Photonics, Hamamatsu City, Japan) were used in the present study to capture, view and score images. As previously reported 12, each slide was scanned using a 20× or 40× objective lens of the NanoZoomer, and the slide code details, the scanning area and the number of focus points for each slide were determined by the user. Slide images were exported to an external storage device in Ndpi format and viewed using NanoZoomer Digital Pathology software (Hamamatsu Photonics, Hamamatsu City, Japan). The NanoZoomer Digital Pathology viewing software was used for capturing and outputting target screenshot as Tiff format for further analysis.

## Results

### The expression and localization of Sox10 in hair follicles changed during hair morphogenesis

**Figure 1:**
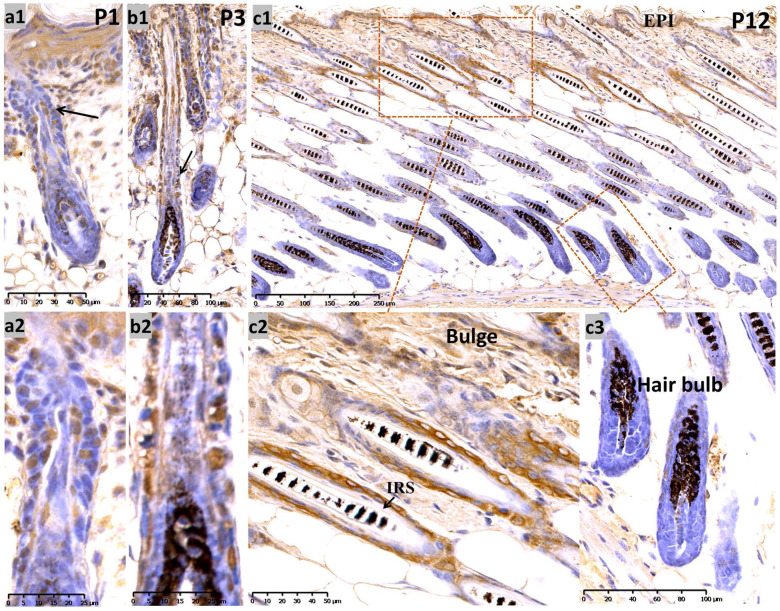
** Expression of Sox10 at P1, P3 and P12, postnatal follicular morphogenesis and anagen of mouse. (a1-b2)** P1 and P3: Sox10 staining was significantly intensified in growing epithelium-derived follicular compartments in a nuclear pattern as displayed by the black arrow head; a2 and b2 represented the magnification inserts of a1 and b1, respectively. **(c1-c3)** at P12, Sox10 expression intensified in the interfollicular epidermis and bulge regions, whereas the hair bulb and IRS stained negative. EPI, epidermis; IRS, inner root sheath. Scale bar: (a1, c2) is 50 µm, (a2, b2) is 25 µm, (b1, c3) is 100 µm, (c1) is 250 µm.

During late embryogenesis (E18.5), the bulb-like thickening of hair peg cells was highly proliferative. Sox10 showed high intensity staining in the basal epithelium and hair peg in a nucleus and peri-nuclear pattern (Supplementary Figure, Figure S1a). We analyzed Sox10 expression during the postnatal period at postnatal day 1 (P1) and P3 and found that Sox10 staining was expressed in growing epithelium-derived follicular compartments in a primarily nuclear pattern **(Figure 1, a-b)**.

At P12** (Figure 1, c1-c3)** (postnatal mid-anagen), intense staining of Sox10 was observed in the interfollicular epidermis, infundibulum and bulge keratinocytes, whereas HFs displayed relatively weaker staining in the bulbar outer root sheath (ORS). The inner root sheath (IRS) and bulbar matrix stained negative at P12.

**Figure 2:**
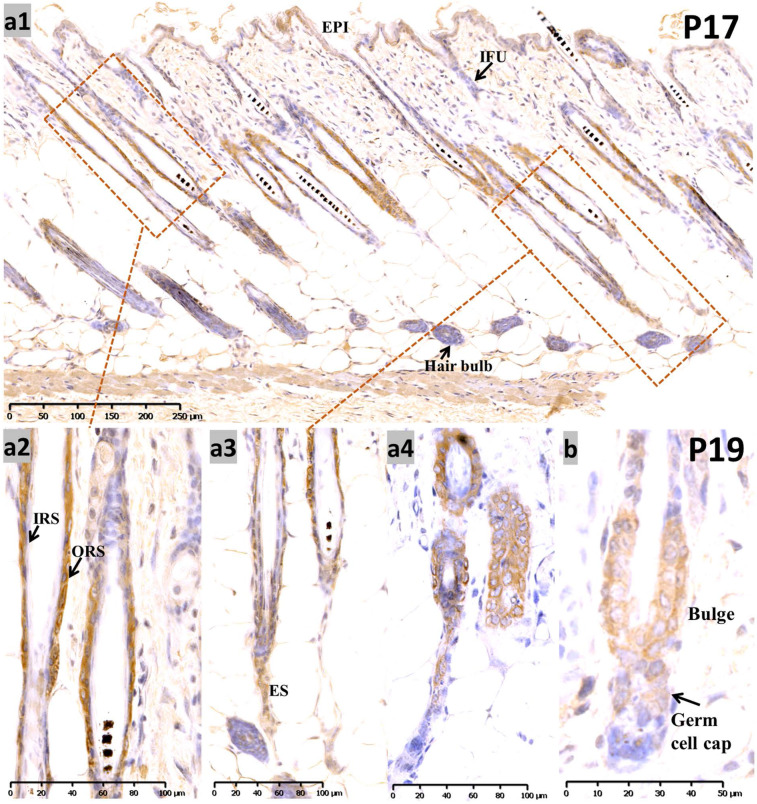
** Expression of Sox10 at P17 and P19, postnatal catagen and telogen of mouse. (a)** During P17, Sox10 staining in the isthmus ORS and regressing ES was significantly intensified **(a1-a3)**, relatively stronger than infundibulum and interfollicular epidermis. Nuclear staining pattern was observed in the regressed epithelial strand and germ capsule in late catagen **(a4)**. **(b)** During telogen (P19), Sox10 staining accumulated in the bulge and germ cell cap regions. EPI, epidermis; IFU, infundibulum; ORS, outer root sheath; IRS, inner root sheath; ES, epithelial strands. Scale bar: (a1) is 250 µm, (a2-a4) is 100 µm, (b) is 50 µm.

At P17, when most of the hair follicles entered catagen after birth, there was intense staining in the apoptotic isthmus ORS and regressing epithelial strands (ES) in mid-catagen** (Figure 2, a1-a3)**, and it gradually retreated from the epithelium until only moderate nuclear staining was observed in the regressed epithelial strand and germ capsule in late catagen **(Figure 2, a4; Figure S1, b1-b3).** We examined the co-staining of Sox10 and Caspase3, a key element of apoptosis during catagen 13. Double labeling with Capase3 further demonstrated the staining pattern of Sox10 during catagen **(Figure S1, g1-g5).** Meanwhile, infundibulum and IRS **(Figure 2, a1-a2)** stained weak during catagen.

During the first postnatal telogen (P19), when the HF regressed to a small dormant structure that appeared as a club hair, Sox10 was localized in the germ cell cap and bulge surrounding the club hair **(Figure 2b)**.

**Figure 3:**
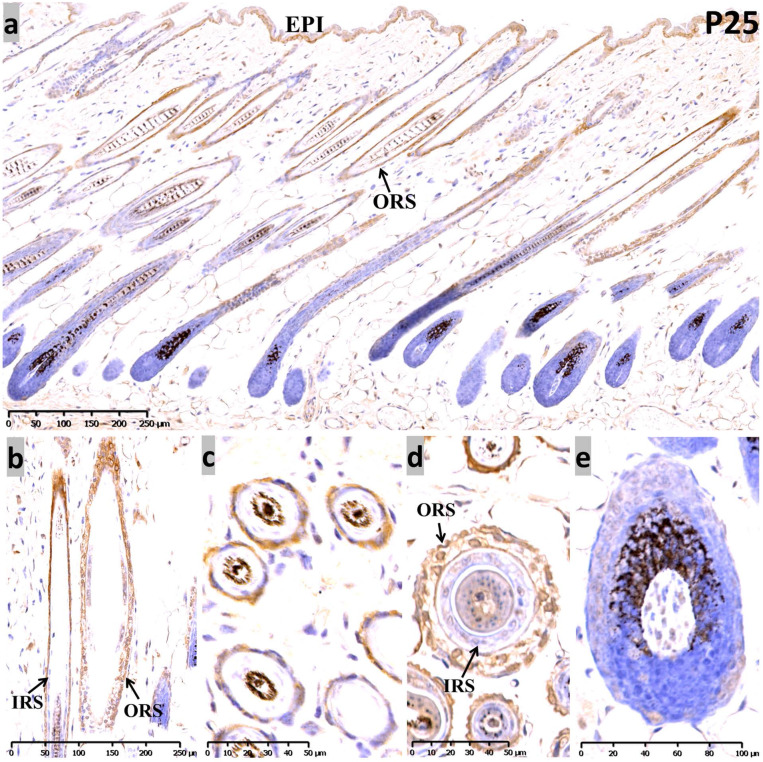
** Expression of Sox10 at P25, postnatal follicular anagen of mouse. (a-c)** We found positive Sox10 expression within the epidermis and ORS. **(b, d)** Note the nucleus staining of inner layer of isthmus ORS and no staining in IRS. **(a, e)** Scattered Sox10 expression was observed in the suprabulbar ORS and bulbar hair matrix keratinocytes. EPI, epidermis; ORS, outer root sheath; IRS, inner root sheath. Scale bar: (a,b) is 250 µm, (c,d) is 50 µm, (e) is 100 µm.

At P25, the sequential anagen phase, moderate Sox10 immunoreactivity was redetected in the epidermis, as well as the whole ORS **(Figure 3)**. Proliferating cells in anagen mouse skin were mostly observed in the epidermis, the ORS and the actively dividing matrix cells of the hair bulb. Interestingly, Sox10 displayed nuclear staining pattern within the inner layer of the isthmus ORS** (Figure 3b, d)**. Notably, positive cytoplasmic staining was observed in the infundibulum and isthmus ORS keratinocytes at anagen (P12 and P25), but Sox10 was prominent in the nucleus in the late-catagen and telogen phase. Negative controls were performed and displayed as Supplementary Figure 4.

### The expression and localization of Sox10 changed during the depilation-induced hair cycle

**Figure 4:**
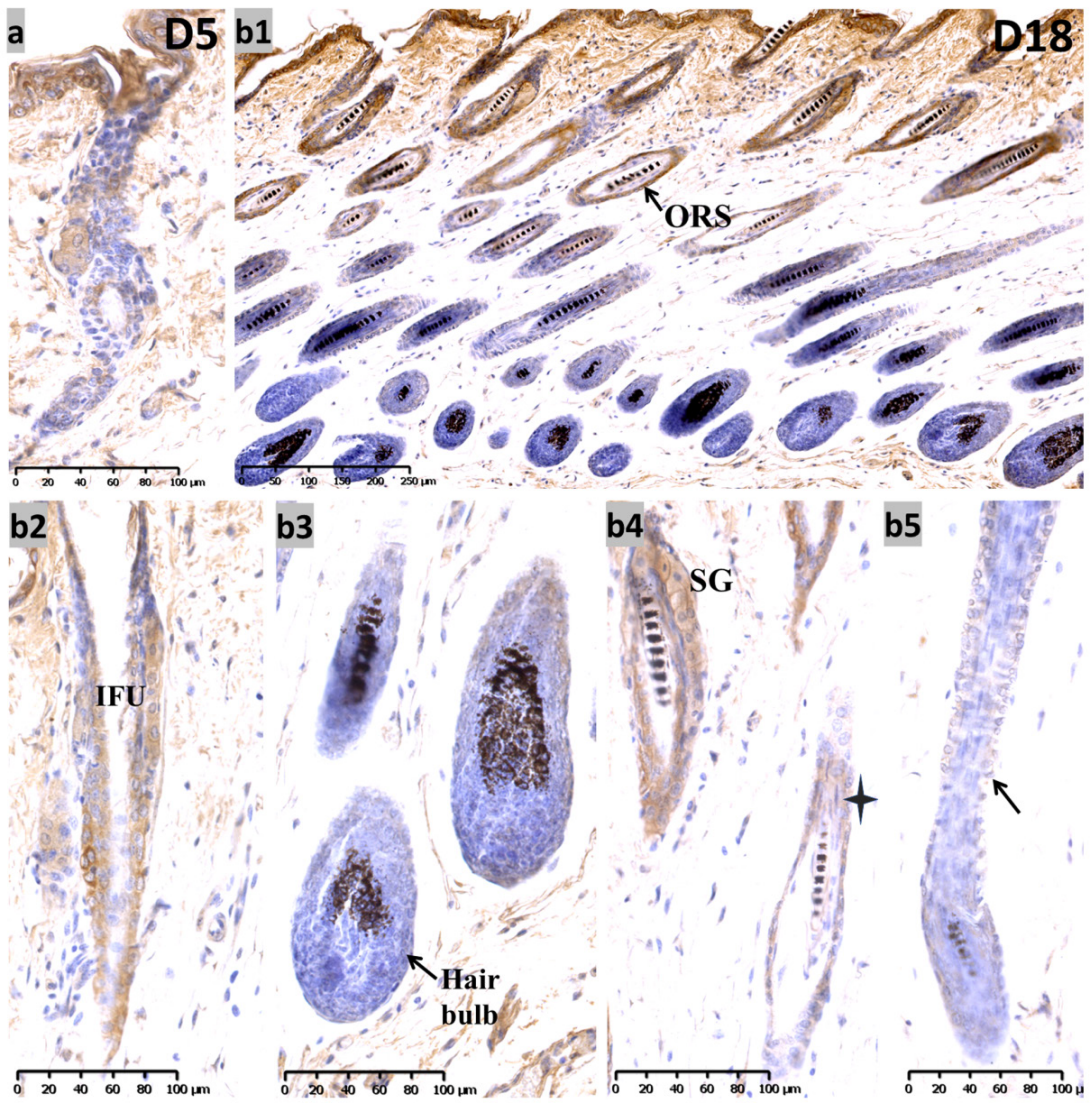
** Expression of Sox10 at depilation-induced day 5 and 18 (anagen IV and catagen). (a)** During depilation-induced anagen (D5), Sox10 staining was intensified in growing epithelium-derived follicular compartments. **(b1-b5)** During depilation-induced catagen (D18), Sox10 expression retreated from the apoptotic ORS regions, while moderate staining was observed in the infundibulum (b2) and isthmus ORS **(star, b4)**, whereas the hair bulb **(b3)** and lower ORS** (black arrowhead, b5)** stained negative. ORS, outer root sheath; IFU, infundibulum; SG, sebaceous glands. Scale bar: (a,b2-b5) is 100 µm, (b1) is 250 µm.

**Figure 5:**
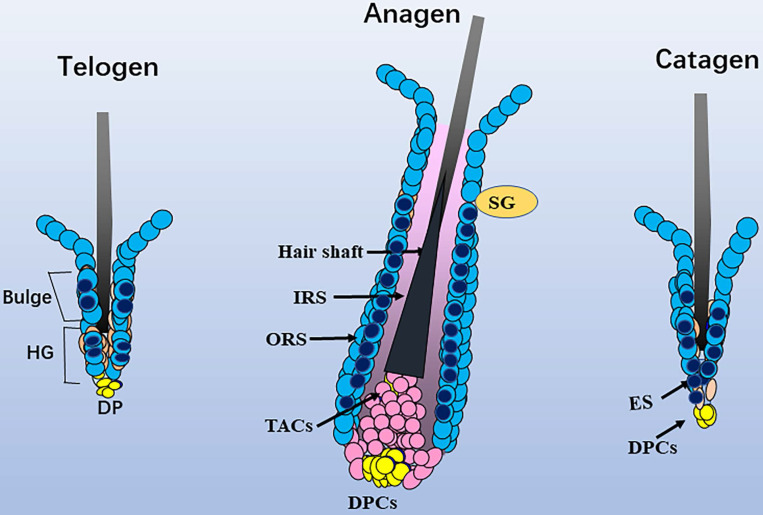
**Schematic diagram of the distribution of Sox10.** In telogen, nucleus Sox10 was mainly distributed in the bulge and germ cell cap regions; at fully developed anagen, nucleus sox10 was showed in the ORS below sebaceous glands, especially the inner layer of ORS; during catagen, Sox10 was observed in the germ capsule and epithelial strand. Light blue cell with dark blue core described mainly nuclear staining of Sox10, and only light blue cell described mainly cytoplasmic staining of Sox10. ORS, outer root sheath; IRS, inner root sheath; HG, hair germ; TAC, transit-amplifying cell; ES, epithelial strand; DP, dermal papilla cells; SG, sebaceous glands.

During the telogen phase (0 days after depilation, D0), nuclear staining for Sox10 was observed in bulge and hair germ cap** (Figure S1, c1-c3)** - an expression pattern that is reminiscent of that at P19. During the anagen II phase (D3)** (Figure S1, d1-d3)**, Sox10 was intensely expressed throughout the interfollicular epidermis and displayed strong immunolabelling in the proliferating strand of keratinocytes in a nuclear pattern. On the fifth day after hair depilation (D5), the hair follicles entered anagen IV. The interfollicular epidermis was thickened and Sox10 showed stronger immunostaining in interfollicular epithelium and infundibulum, the hair germ and bulge regions **(Figure 4a)**. As the follicles approached the catagen phase (18 days post depilation), Sox10 staining gradually retreated from the ORS, stronger Sox10 staining was observed in the infundibulum and upper ORS than in the apoptotic lower ORS **(Figure 4, b1-b5)**.** Figure 5** displayed the schematic diagram of the distribution of Sox10 during telogen, anagen and catagen.

### The co-localization of Sox10 with hair follicular stem cell markers during hair morphogenesis

The bulge compartment contains slowly cycling stem cells that are important for replenishing the pool of follicle cells during hair cycling. Stem cells in the bulge express high levels of CD34 14. Double labeling with CD34 demonstrated that Sox10 is expressed in both the outer and inner layers of bulge hair follicle stem cells, whereas CD34 staining mainly intensified in the outer layer during telogen **(Figure S1, e1-e4)**.

Keratin14 (K14) was expressed in the basal epidermis and outer root sheath of hair follicles 15. Co-staining with K14 revealed that Sox10 is expressed in most epithelial compartments including ORS during anagen, the nuclei of regressing epithelial compartment during catagen, the bulge and hair germ regions during telogen** (Figure S2)**.

Previous study revealed that Sox10 is necessary for maintenance of melanocyte stem cells and committed melanoblasts 16. The results of double labeling of Sox10 with CD44 (melanoma stem cell marker) revealed that specific nucleus Sox10 expressed in melanocyte stem cell within the bulge **(Figure S3, a1-b4)**, anagen hair bulb **(Figure S3, b5-b7)** and epithelial strand in catagen **(Figure S3, c1-c4)**.

## Discussion

We provide evidence that Sox10 is expressed in hair follicle structures, mainly including the infundibulum, hair bulge, hair germ cells and ORS. Sox10 expression was dynamically accompanied by hair morphogenesis and hair cycling. In anagen, nuclear Sox10 is widely distributed in the epithelium, particularly in the interfollicular epidermis, the rapidly growing strand of keratinocytes, ORS and HFSCs regions at anagen. However, Sox10 staining was negative in IRS and DPs.

In catagen, immunostaining for Sox10 was associated with dynamic changes in the hair follicle structure. During mid-catagen, it was more intense in the apoptotic middle ORS and lower epithelial strands. When late catagen progressed, it retreated from the epidermis and infundibulum, and accumulated in the permanent bulge and the regressed epithelial strand. In telogen, it was mainly localized in the two populations with stem or progenitor qualities, the hair bulge and germ region. These results suggest that Sox10 expression may be involved in HF cycling and restricted to the HFSC region as the nascent follicle retreats back up the dermis throughout the catagen-telogen transition. During early anagen, Sox10 intensified in the hair follicle strand of keratinocytes, which is involved in the induction of the hair cycle. Furthermore, previous reports have confirmed the role of other Sox family transcription factors Sox9, Sox13 and Sox21 in follicular morphogenesis and stem cell differentiation 17-19.

Previous studies have shown that Sox10 expression could direct neural crest cell differentiation into diverse lineages such as melanocytes, glia, and cartilage 3. Neural crest cells are involved in the earliest stages of hair follicle development and neural crest-derived hair follicle cells may have properties in common with multipotent neural crest cells 20. Stephanie Watson reported positive Sox-10 staining in dermoids and limbus basal epithelium stem cells, as well as in human hair follicles, the ORS and bulb, and in sebaceous and sweat glands 21. These results are consistent with our findings in murine follicles. Despite numerous studies focusing on the neural crest cell-HFSC transition 22-23, the relationship between resident neural crest cells and HFSCs in hair follicle biology requires further investigation. Sox10 is static relative to HF cycling and restricted to the bulge and infundibulum region as the nascent follicle retreats back up the dermis during catagen-telogen transition; Sox10 is most densely nuclear in telogen (D0 and P19). The constant presence of Sox10 in hair stem cells or their progenitor during both hair morphogenesis and depilation-induced hair cycling suggests that Sox10 may function as a novel niche modulator of HFSCs and it may function in stem cell quiescence. Further, co-staining of Sox10 and CD44 reveals that Sox10 exhibits divergent functional roles in murine melanocyte stem cells and hair pigmentation.

HFSCs localized in various epidermal compartments have been shown to play a role in skin cancer pathogenesis, and irreversible damage to epithelial stem cells of the hair follicle in their immunologically privileged niche lies at the heart of scarring alopecia 24-26. Although a role of Sox10 in diverse stem cells has been identified, its characteristics with regard to HFSC control and hair morphogenesis are not fully understood. Our study is the first to provide direct evidence regarding Sox10 expression in murine hair morphogenesis and cycling. This opens up new research directions to further explore mechanisms in hair growth and development, and provides new ideas for the pathogenesis of hair-related diseases and tumours of hair follicle origin.

## Supplementary Material

Supplementary figures.
