# Supplementary material for: Expression and localization of Sox10 during hair follicle morphogenesis and induced hair cycle
Source: Int J Med Sci. 2021 Aug 13;18(15):3498–505. doi: 10.7150/ijms.60728 (PMC8436095; doi:10.7150/ijms.60728)
Supplement: Supplementary file 1 — Supplementary figures. [file ijmsv18p3498s1.pdf]

Figure S1

(a-d) Expression of Sox10 at E18.5, postnatal day 17, and depilation-induced day 0 and day 3

(a1-a3) During late embryogenesis E18.5, Sox10 intensified in the basal epidermis and hair peg in nucleus and peri-nucleus pattern. **(b1-b3)**: During late catagen at P17, nucleus Sox10 is restricted to the lower regressing epithelial strand, and appeared within the regenerated bulge region. **(c1-c3)**: During telogen (D0), nucleus staining for Sox10 was observed in bulge and germ cell cap. **(d1-d3)**: During anagen II (D3), Sox10 was intensely expressed throughout the interfollicular epidermis and displayed strong immunolabeling in the proliferating strand of keratinocytes in a nucleus pattern. Sox10 staining showed green fluorescence. Blue is DAPI nucleus stain. *SG* sebaceous glands.

(e-g) Double staining for Sox10 and CD34, Ki67 and Caspase3 during hair follicle cycle

(e1-e4) Double staining for Sox10 (green) and CD34 (red) during telogen (P19) revealed that nucleus Sox10 expressed in the whole hair bulge and hair germ, while CD34 mainly stained the outer layer of bulge;

(f1-f4) Double staining for Sox10 (green) and Ki67 (red) at P25 showed scattered Ki67 staining and negative Sox10 staining within the anagen hair bulb;

(g1-g5) Double staining for Sox10 (green) and Caspase3 (red) during catagen (P17) showed that nucleus Sox10 stained in the regenerated bulge region and lower regressing epithelial strand during catagen follicles.

Scale bar: (a1-c3, e1-e4, g1-g5) is 100 μ m, (d1-d3) is 250 μ m, (f1-f4) is 50 μ m.

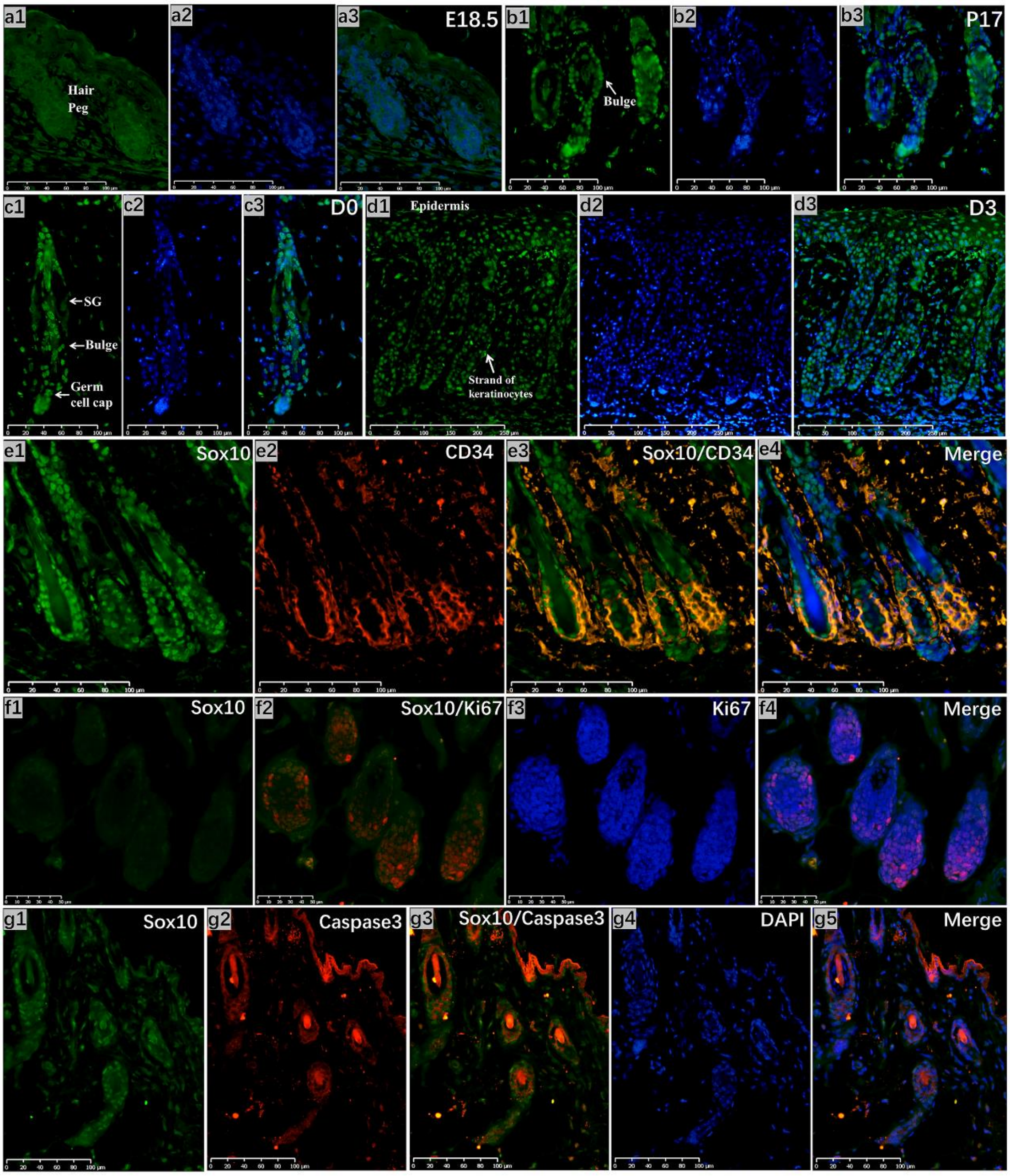
a1
a2
a3
E18.5
Hair Peg
b1
Bulge
b2
b3
P17
c1
SG
Bulge
Germ cell cap
c2
c3
D0
d1
Epidermis
Strand of keratinocytes
d2
d3
D3
e1
Sox10
e2
CD34
e3
Sox10/CD34
e4
Merge
f1
Sox10
f2
Sox10/Ki67
f3
Ki67
f4
Merge
g1
Sox10
g2
Caspase3
g3
Sox10/Caspase3
g4
DAPI
g5
Merge

Figure S2

Double staining for Sox10 and Keratin14 during hair follicle cycle

(a1-a4) Double staining for Sox10 (green) and Keratin14 (red) during anagen (P25). Sox10 stained the whole ORS, whereas K14 mainly stained the isthmus ORS;

(b1-b10) Double staining for Sox10 (green) and Keratin14 (red) during early catagen (b1-b6) and late catagen (b7-b10) (P17);

(c1-c4) Double staining for Sox10 (green) and Keratin14 (red) during telogen (P19)

Scale bar: (a1-b6) is 250 μ m, (b7-c4) is 100 μ m.

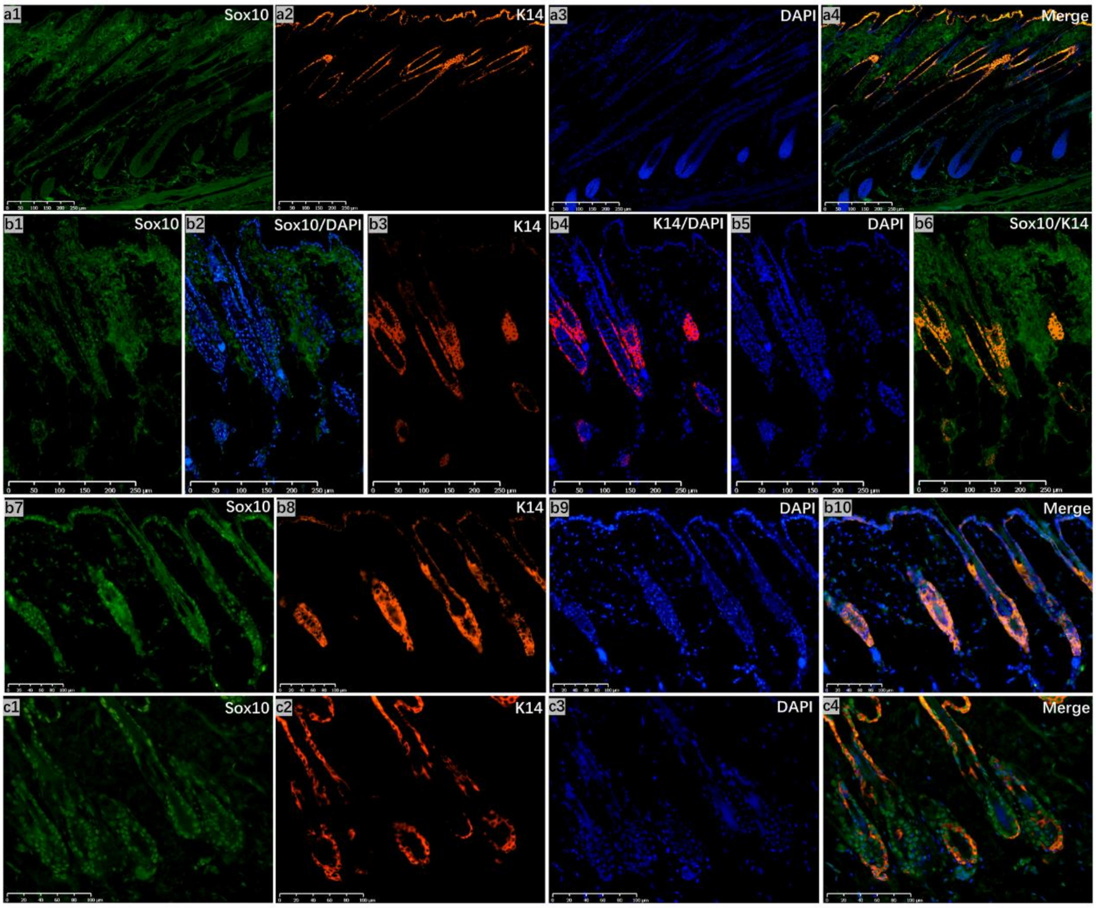

Figure S3

Double staining for Sox10 and CD44 during hair follicle cycle
(a1-a4) Double staining for Sox10 (green) and CD44 (red) during telogen (P19)
(b1-b4) Double staining for Sox10 (green) and CD44 (red) within bulge regions during anagen (P25)
(c1-c4) Double staining for Sox10 (green) and CD44 (red) within the epidermis and ORS during anagen (P25). CD44 showed cytoplasmic staining in the bulge region and bulbar ORS (white arrowhead). Magnified co-staining of Sox10 and CD44 within the bulge regions were shown in **(b1-b4).**
(d1-d4) Double staining for Sox10 (green) and CD44 (red) during catagen (P17)
Scale bar: (a1-a4, d1-d4) is 100 μ m, (b1-b4) is 50 μ m, (c1-c4) is 250 μ m

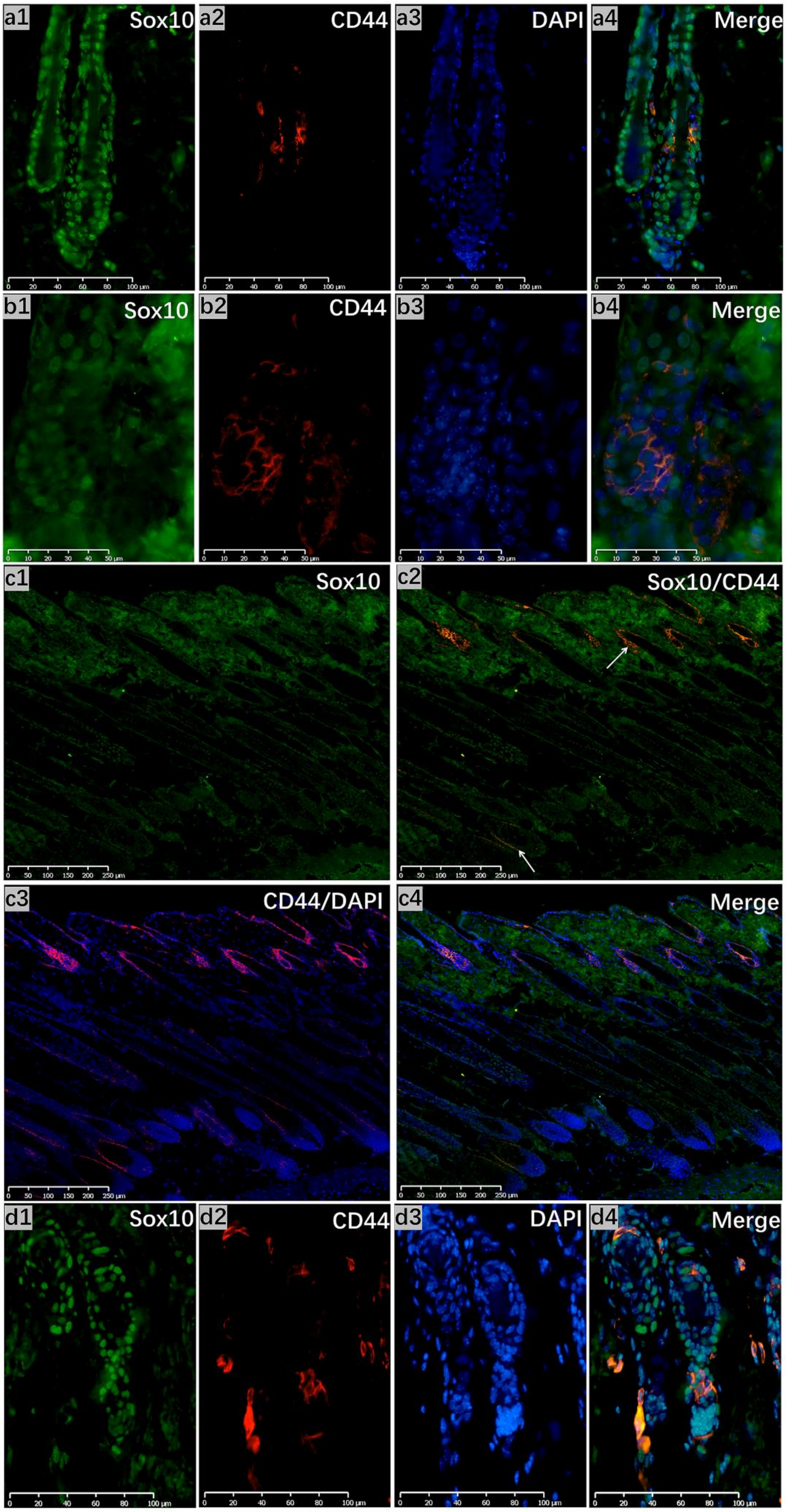
a1
Sox10
a2
CD44
a3
DAPI
a4
Merge
b1
Sox10
b2
CD44
b3
b4
Merge
c1
Sox10
c2
Sox10/CD44
c3
CD44/DAPI
c4
Merge
d1
Sox10
d2
CD44
d3
DAPI
d4
Merge

Figure S4:

Negative controls with no primary antibody but including secondary antibody

(a) Negative controls for immunohistochemical study

(b) Negative controls for immunofluorescent study

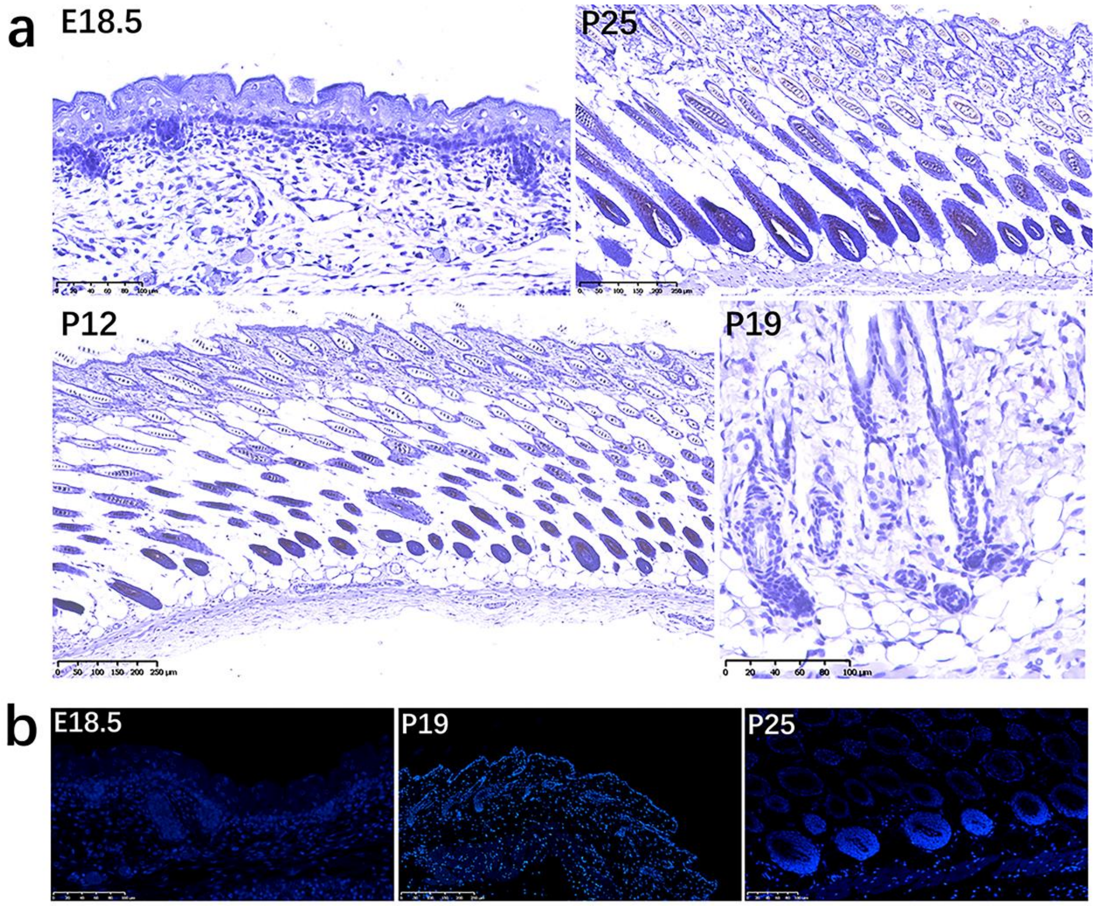

Supplementary File 5: Antibody information

Source of antibody used in the supplementary figure experiments: The primary antibody of anti-CD34 is rabbit monoclonal anti-CD34 antibody（Abcam, ab81289）. The primary antibody of anti-CD44 is rabbit monoclonal anti- CD44 antibody（Abcam, ab189524）. The primary antibody of anti-cytokeratin 14 is rabbit monoclonal anti-cytokeratin 14 antibody（Abcam, ab181595）. The primary antibody of anti-Ki67 is rabbit monoclonal anti-ki67 antibody（Abcam, ab16667）. The primary antibody of anti- Caspase3 is rabbit polyclonal anti- Caspase3 antibody（Abcam, ab13847）.The secondary antibody in double immunofluorescent staining is 1/1000 Alexa Fluor®488 conjunctated goat polyclonal secondary antibody to mouse IgG (Abcam, ab150113) or goat anti-rabbit IgG H&L (Alexa Fluor® 555) (Abcam, ab150078).
